# Primaquine for *Plasmodium vivax* radical cure: What we do not know and why it matters

**DOI:** 10.1016/j.ijpddr.2020.12.004

**Published:** 2021-01-24

**Authors:** Jean Popovici, Kieran Tebben, Benoit Witkowski, David Serre

**Affiliations:** a Malaria Molecular Epidemiology Unit, Institut Pasteur du Cambodge, Phnom Penh, Cambodia; b Malaria Translational Research Unit, Institut Pasteur, Paris & Institut Pasteur du Cambodge, Phnom Penh, Cambodia; c Institute for Genome Sciences, University of Maryland School of Medicine, Baltimore, USA

## Abstract

*Plasmodium vivax* radical cure requires the administration of a blood schizonticide for killing blood-stage parasites and the addition of a drug able to kill hypnozoites, the dormant parasite stages residing in the liver of infected patients. All drugs used clinically for killing hypnozoites are 8-aminoquinolines and among them, primaquine has been at the forefront of *P. vivax* case management for decades. We discuss here the possible factors that could lead to the emergence and selection of *P. vivax* primaquine resistant parasites and emphasize on how a better understanding of the mechanisms underlying primaquine treatment and hypnozoite biology is needed to prevent this catastrophic scenario from happening.

## Graphical abstract

**Figure:**
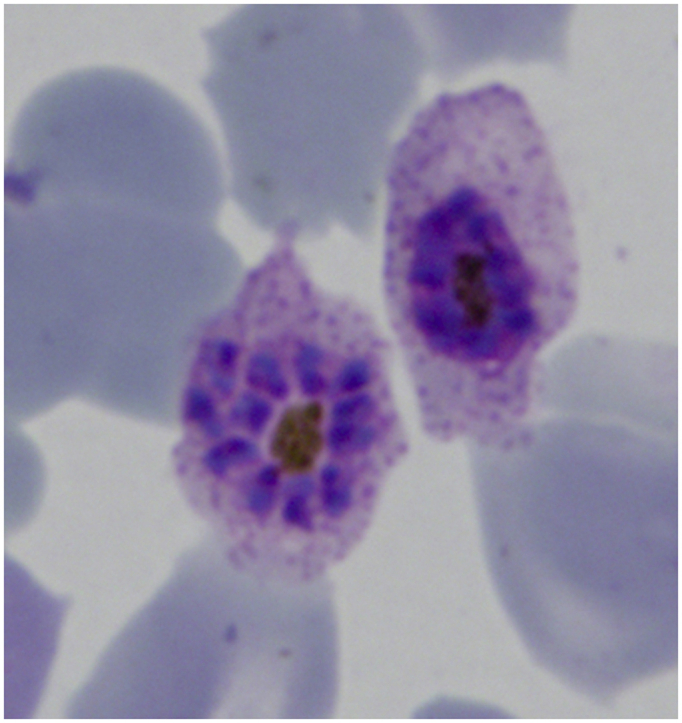

## 1 Introduction

*Plasmodium vivax* is the human malaria species with the widest geographic distribution and more than three billion people live within the *P. vivax* transmission limits (Battle et al., 2019). It is responsible for the majority of malaria cases outside Africa. Considered as benign for decades, it is now clear that *P. vivax* malaria is a significant cause of morbidity and mortality in endemic populations (Baird 2013; Genton et al., 2008; Douglas et al., 2014). *P. vivax* harbors biological features that greatly complicate malaria control and elimination, including a unique developmental stage in which some sporozoites develop into hypnozoites and remain dormant in the liver for weeks or months, before reactivating and causing relapse infections (Mueller et al., 2009; Krotoski 1985; White and Imwong 2012).

The existence of a unique dormant stage of *Plasmodium vivax* has been rigorously characterized in studies of volunteer infections and WWII soldiers in the first half of the 20th century (White and Imwong 2012) but the mechanisms triggering hypnozoite activation remain unknown. Studies of patients treated with *P. vivax* for neurosyphilis revealed that different strains of *P. vivax* relapsed at different intervals and highlighted differences between “tropical strains”, with early and frequent recurrence, and “temperate” strains, with a long latency (White 2011; Lysenko et al. 1977), suggesting that the timing of the hypnozoite reactivation was genetically encoded. Alternatively, external stimuli, such as infections with other pathogens (Shanks and White 2013), have also been proposed to influence hypnozoite reactivation. In particular, in many areas where *P. vivax* and *Plasmodium falciparum* are co-endemic, *P. vivax* blood stage infections can often be detected following treatment for *P. falciparum* malaria (Hossain et al., 2020). Some authors have suggested that *P. vivax* hypnozoite activation could be triggered by *P. falciparum* infection (Snounou and White 2004) although this could also simply reflect the frequent relapses of individuals living in co-endemic areas (Popovici et al., 2018).

Current studies of *P. vivax* relapse rely primarily on travelers or military personal who are exposed to vivax malaria for a limited period before returning to a malaria-free area. This approach allows to rigorously study relapse patterns while avoiding confounding effects of reinfections. However, such studies might not adequately recapitulate the relapses of individuals living in endemic countries who are exposed to numerous infectious bites over extended periods of time. Using patient relocation in a no-transmission area and comprehensive genetic analyses, we recently showed that relapses in Cambodian individuals are much more complex and prevalent than previously thought, with at least 60% of the patients studied relapsing within a two-month period (Popovici et al., 2018). In particular, this study suggested that hypnozoites reactivate constantly and that individuals in endemic countries likely carry many hypnozoites. Several studies have reached similar conclusions and modeling analysis suggest that in some areas, as high as 96% of *P. vivax* episodes are due to relapses rather than mosquito inoculations (Adekunle et al., 2015; Commons et al., 2020; Robinson et al., 2015).

This high prevalence of hypnozoites is extremely worrying for vivax malaria control since i) it will complicate parasite elimination campaigns (as hypnozoites are not affected by blood-stage antimalarials typically used for treating patients) and ii) dormant parasites facilitate the spread of *P. vivax* over long distances and reintroduction of the disease to malaria-free areas (as they are carried by infected but apparently healthy individuals). Therefore, relapses constitute the main challenge of *P. vivax* control and elimination efforts. Radical cure, the complete elimination of all parasites from a patient, is difficult since primaquine, the only WHO-approved drug targeting hypnozoites, has important limitations, including side-effects in G6PD-deficient patients (Baird 2015) and poor patient adherence (due to the long treatment usually over 14 days, see below). Primaquine is an 8-aminoquinoline, a class of molecule displaying antimalarial properties developed in the 1920s, first with plasmochin and some 30 years later with primaquine (Baird 2019). Initially developed for their blood schizonticide properties (killing blood-stages parasites), it was rapidly noticed that those drugs were also effective at preventing relapses. Equally rapid was the acknowledgement of the hemolytic toxicity of those drugs in a number of patients but it was only in the 1950s that G6PD deficiency was identified as at the origin of this toxicity (Baird 2015). Since then, the only other drug that has achieved registration (by the United States Food and Drug Administration and the Australian Therapeutic Goods Administration) for anti-hypnozoite activity is tafenoquine, also an 8-aminoquinoline with similar hemotoxicity in G6PD-deficient patients but with the operational advantage of being single-dose, improving compliance (Tan and Hwang 2018; Rueangweerayut et al., 2017). However, tafenoquine has a long half-life remaining in the blood for several days after single-dose, which prevents the possible interruption of treatment in case of acute hemolysis and could results in more severe side effects (compared to primaquine that can be stopped rapidly if the patient shows any complications). Until widespread availability of a rapid, cheap and accurate point-of-care quantitative assay to measure an individual's G6PD activity (currently under validation), the standard of care for preventing *P. vivax* relapses remains primaquine in most malaria-endemic areas where G6PD deficiency can be common (in many endemic countries, primaquine is not even routinely provided due to fear of side effects).

Here, we briefly review our current knowledge on primaquine mode of action and the factors influencing its efficacy, the uncertainties related to adequate regimen for effective treatment and the challenges in evaluating proper therapeutic efficacy. Finally, we discuss how those issues may influence the emergence and selection of primaquine-resistant parasites.

## 2 Mode of action of primaquine

The mode of action (MoA) of primaquine has remained elusive for decades. Primaquine is a pro-drug that is required to be metabolized for the generation of molecules displaying activity against hypnozoites (Pybus et al., 2013). The metabolism is rapid following drug intake with primaquine reaching peak levels in plasma within 2–3 h then declining rapidly with a terminal phase elimination half-life of 7.1 ± 1.6 h (Mihaly et al., 1984). The major plasma metabolite detected following primaquine administration is carboxy-primaquine which reaches peak levels tenfold higher than primaquine within 3–12 h after dosing (Mihaly et al., 1984). Carboxy-primaquine results from oxidative deamination of primaquine involving monoamine oxidase A (MAO-A) and its concentration in plasma is sometimes used as a proxy of proper primaquine administration (Constantino et al., 1999). Carboxy-primaquine, however, does not show any antimalarial activity and active metabolites result from other metabolic pathways (Constantino et al., 1999).

**Fig. 1:**
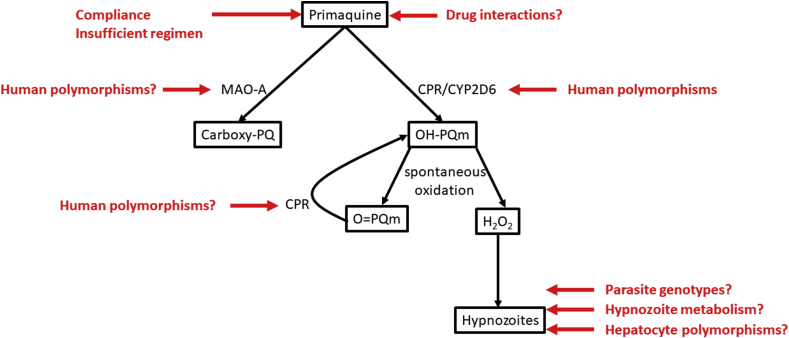
Schematic representation of the different factors and the key questions that could, in theory, affect the emergence and selection of primaquine resistant *Plasmodium vivax* parasites. Compliance to several days of treatment is a challenge and incomplete course (or insufficient regimen) will lead to sub-optimal treatment. Some drugs might inhibit the proper metabolism of primaquine thereby reducing the generation of active compounds (hydoxy-primaquine: OH-PQm). Primaquine metabolism into active compounds requires the involvement of at least two enzymes, CPR and CYP2D6, for the generation of hydrogen peroxide and hypnozoite killing. Human polymorphisms in CYP2D6 and perhaps in CPR can affect proper metabolism of primaquine resulting in improper efficacy. Conversely, primaquine can be funneled into oxidative deamination pathway with the involvement of at least MAO-A. Could human polymorphisms in MAO-A lead to a decrease in primaquine engaged in the CPR/CYP2D6 pathway and result in improper efficacy? Some parasite genotypes might already be less susceptible to primaquine than others: could tolerant alleles be selected upon primaquine treatment? Are there developmental stages of hypnozoites more prone to cope with primaquine damages or infected hepatocytes with reduced primaquine metabolism?.

Several studies have suggested that the MoA of primaquine was due to the generation of reactive oxygen species (ROS) through the cycling of hydroxylated primaquine metabolites (Vale et al. 2009; Pybus et al., 2013; Vásquez-Vivar and Augusto 1992). A recent study performed on *P. falciparum* hepatic stage has provided strong evidence for such mechanism (Camarda et al., 2019). One can hypothesize that this pathway is also responsible for the activity against *P. vivax* hypnozoites, although a formal demonstration is still lacking. In the proposed MoA, activity of primaquine results from a two-step process: first primaquine is hydroxylated into hydroxyl-primaquine metabolites (OH-PQm) by the complex made of the NADPH cytochrome P450 oxidoreductase (CPR) and the cytochrome P450 2D6 (CYP2D6). OH-PQm are then spontaneously oxidized in quinoneimines (O=PQm) producing H_2_O_2_. NADPH-dependent reduction of O=PQm by CPR recycles the metabolites back to their hydroxylated forms generating successive cycles of H_2_O_2_ production. It is then H_2_O_2_ that directly kills parasites (Fig. 1).

## 3 Drug-drug interactions for primaquine anti-hypnozoite efficacy

Early on, it was observed that primaquine efficacy at preventing relapses was enhanced when administered with blood schizonticidal drugs devoid of anti-hypnozoite activity by themselves (such as quinine or chloroquine) (Baird 2019). The same observation was made for tafenoquine: the effective dose for killing hypnozoites of *Plasmodium cynomolgi* (often used as surrogate model for *P. vivax* infections) was lowered when tafenoquine was associated with other antimalarials (Dow et al., 2011). However, a recent clinical trial has shown that primaquine given after administration of a rapidly eliminated blood schizonticide (artesunate) was as effective as when given concomitantly with artesunate-pyronaridine or dihydroartemisinin-piperaquine indicating that primaquine by itself provided adequate anti-hypnozoite activity (Nelwan et al., 2015). Differences in dosage and drugs used in those different studies might explain the discrepancy observed and more work is needed to understand the possible interactions between antimalarial drugs and the mechanisms involved. Conversely, it is possible that drug interactions lead to reduced hypnozoitocidal efficacy of primaquine (or tafenoquine). So far, studies have shown that primaquine displays adequate anti-hypnozoite activities when provided with most blood-stage antimalarials such as chloroquine, dihydroartemisinin-piperaquine, artesunate-pyronaridine or artesunate-amodiaquine (Pasaribu et al., 2013; Taylor et al., 2019; Commons et al., 2018; Nelwan et al., 2015). There was initially some concerns regarding lumefantrine administered with primaquine as lumefantrine is an inhibitor of CYP2D6 in vitro (White et al. 1999) and is a schizonticide used in combination with artemether in many endemic countries. A recent meta-analysis showed that primaquine administered with artemether-lumefantrine did reduce the risk of recurrence within 42 days by 80% compared to artemether-lumefantrine alone, indicating that a possible lumefantrine inhibition of CYP2D6 was not sufficient to completely abolish the action of primaquine (Commons et al., 2019). In a study conducted in Ethiopia, the efficacy of primaquine at preventing relapses over a year of follow-up was similar between patients treated concomitantly with chloroquine or artemether-lumefantrine indicating that CYP2D6 inhibition by lumefantrine is not clinically relevant (Abreha et al., 2017).

Concerning tafenoquine, it has mainly been administered in clinical trials either alone or in combination with chloroquine (Llanos-Cuentas et al. 2019; Llanos-Cuentas et al., 2014), and its interactions with most other antimalarials are unknown. However, it was recently reported that tafenoquine administered with dihydroartemisinin-piperaquine did not prevent *P. vivax* relapses as it does when administered with chloroquine (Baird et al., 2020). Whether poor tafenoquine efficacy in this study resulted from drug interaction with dihydroartemisinin-piperaquine or from under-dosing of tafenoquine is unknown and warrants further investigations.

## 4 The total dose effect

Another old observation is that the efficacy of primaquine treatment appears to be influenced by the total dose administered, rather than the duration of the treatment or the maximal concentration achieved in the blood (a phenomenon referred to as the total dose effect) (Schmidt et al., 1977; Clyde and McCarthy 1977). This clearly contrasts with most antimalarials (if not all) where the critical parameter to achieve parasite elimination is whether the drug concentration in the blood is maintained sufficiently high, over the time necessary to kill all parasites. One hypothesis to explain the total dose effect of primaquine is that irreversible and cumulative damages are caused to hypnozoites by primaquine metabolites and, due to their reduced metabolism, these dormant parasites are not able to cope with such damages. This total dose effect offers the possibility to obtain the same efficacy using lower doses delivered over longer period of time (e.g., 14 days), thereby reducing hemolytic toxicity (however, see below our discussion on compliance).

## 5 Factors affecting primaquine activity

A number of human and parasite factors are suspected to be involved in modulating primaquine effectiveness at killing hypnozoites. Some are well known and undisputed (compliance to treatment), while others, such as human genetic polymorphisms, are just beginning to be unraveled. In addition, factors such as host immunity probably contribute to efficient hypnozoitocidal activity by primaquine although no proper evidence has yet linked primaquine efficacy with host immunity.

### 5.1 Adherence to treatment

Compliance to primaquine treatment is a main factor influencing drug efficacy. While there has been a number of primaquine regimens described in the literature in the past 70 years, they all share the feature of being spread over several days, making adherence to treatment very challenging. The current WHO guidelines for primaquine treatment are a 14-day regimen of 0.25–0.5 mg/kg/day, depending on the geographic origin of the parasite (see below) (WHO 2015). Because malaria-related symptoms usually resolve within 24–72 h, it is challenging to convince patients to be compliant to a treatment that will last much longer, especially in low-resource settings where treatments often come with a significant monetary cost for individuals. Several studies have shown that when primaquine administration is not supervised, its effectiveness is much lower than when administered under directly observed therapy (Takeuchi et al., 2010; Douglas et al., 2017; Abreha et al., 2017). An alternative to poor adherence observed in the 14-day regimen is to provide the same total dose but spread over shorter duration (leveraging the total dose effect described above). Two recent randomized controlled clinical trials have shown that 1 mg/kg/day over 7 days was non-inferior to 0.5 mg/kg/day over 14 days at preventing *P. vivax* relapses and was relatively well tolerated in G6PD normal patients (Taylor et al., 2019; Chu et al., 2019). Halving the duration of primaquine regimen would be a major improvement compared to the 14-day therapy for keeping patients compliant, although the higher dose may increase the risk of hemolysis in G6PD deficient individuals (and in heterozygous females). Whether National Malaria Control Programs in areas where G6PD deficiency is common will endorse such regimen is currently unknown.

### 5.2 Human genetic polymorphism

As mentioned above, primaquine is a pro-drug that needs to be metabolized to generate active molecules through the activity of at least two enzymes, CYP2D6 and CPR (Camarda et al., 2019). Many variants are described in human populations for those enzymes and pharmacogenomics studies have shown that different common polymorphisms can increase or decrease enzymatic activity (Zhou et al. 2017). Polymorphisms leading to reduced enzyme activity might lead to impaired metabolism of primaquine and cause treatment failure. This has been recently demonstrated for some of the CYP2D6 polymorphisms (Baird et al., 2018; Bennett et al., 2013).

#### 5.2.1 CYP2D6

More than 113 common alleles have been identified through a combination of single nucleotide (SNVs) and copy number variants (CNVs) (Zhou et al. 2017; Nofziger et al., 2020). Compared to the “normal” CYP2D6 activity of “extensive metabolized (EM)”, some genotypes lead to increased CYP2D6 activity and the individuals being “ultra-rapid metabolizers (UM)” (Nofziger et al., 2020). However, the problematic genotypes, with regards to primaquine treatment, are those responsible for reduced or no CYP2D6 activity (intermediate (IM) or poor metabolizers (PM), respectively) as they result in sub-standard metabolism of primaquine and lower production of active metabolites (Pybus et al., 2013; Nofziger et al., 2020; Spring et al., 2019). Indeed, several studies have demonstrated increased risk of *P. vivax* relapse in IM or PM patients treated with primaquine, indicating a need to incorporate the patient genotype at this gene into the treatment recommendations (Bennett et al., 2013; Baird et al., 2018). Regional differences in the frequency of CYP2D6 alleles have been described but the data remains sparse for malaria endemic areas and there is a critical need to better characterize the distribution of these genetic variations to rigorously evaluate their role in primaquine treatment failures (Zhou et al. 2017; Gaedigk et al., 2017).

#### 5.2.2 CPR

CPR activity is important for its direct role in primaquine metabolism, but also through its interactions with CYP2D6. Complete deficiency of CPR function is rare in humans, leading to congenital adrenal hyperplasia, disrupted steroid biosynthesis and impaired sexual development (Hart and Zhong 2008). However, a number of polymorphisms affecting CPR function have been characterized, some directly affecting the reduction of quinone drugs (Hart and Zhong 2008; Velazquez et al., 2019). The consequences of these CPR polymorphisms appear to be drug-specific and are poorly characterized, but we would expect that some might reduce primaquine metabolism and could influence drug efficacy. In addition, some CPR variants specifically reduce CYP2D6 activity (Pandey and Sproll 2014): A287P and R457H, when occurring together, lead to absence of CYP2D6 activity (similar to PM phenotype) (Sandee et al., 2010) while A287P alone reduces CYP2D6 activity to 25% of wild-type (Sandee et al., 2010). Understanding how these variants influence primaquine metabolism on their own and in combination with CYP2D6 polymorphisms is imperative.

#### 5.2.3 MAO-A

Primaquine also undergoes oxidative deamination by monoamine oxidase A (MAO-A), leading to carboxy-primaquine, a metabolite with no anti-parasitic activity (Constantino et al., 1999). However, this pathway is relevant for primaquine radical cure as increased MAO-A activity could, in theory, decrease the amount of primaquine available for CYP2D6 metabolism into active metabolites and therefore the concentration of anti-hypnozoite compounds. Alternatively, polymorphisms that reduce MAO-A activity might increase anti-parasitic activity if the drug is instead funneled to the CPR/CYP2D6 pathway and generate more active metabolites. Studies on MAO-A polymorphism and association with phenotypes are scarce. Nevertheless, both at the coding sequence level (Hotamisligil and Breakefield 1991) and at the promoter level (Sabol et al. 1998), some polymorphisms of MAO-A are described and affect the enzyme activity. Very few studies have evaluated the consequences of MAO-A polymorphisms on primaquine metabolism (Ariffin et al., 2019). MAO-A polymorphisms cluster geographically among human populations (Balciuniene et al., 2001; Gilad et al., 2002) but information on populations living in vivax-endemic areas are lacking. Whether polymorphism in MAO-A affect anti-hypnozoite outcomes of primaquine treatment is unknown.

### 5.3 Dose of primaquine and parasite genetic background

The current WHO guidelines for primaquine treatment recommend a 14-day regimen of 0.25–0.5 mg/kg/day, depending on the geographic origin of the parasite (WHO 2015). It is indeed believed that parasites originating from tropical areas, especially South East Asia and Pacific Islands are intrinsically less susceptible to primaquine than isolates from temperate areas. Those assumptions are derived from early investigations showing that higher doses of 8-aminoquinoline regimens were necessary to cure *P. vivax* experimental infections with the Chesson strain (an isolate originating from Papua New Guinea) than with the St-Elizabeth strain (whose origin is not clearly established) (Ehrman et al. 1945; Baird 2019). This implies that there are genetically encoded variations in susceptibility to primaquine among *P. vivax* populations. However, caution must be taken as those early investigations were not designed to rigorously test for differences in *P. vivax* drug susceptibilities according to the geographic origin of isolates and these conclusions were extrapolated from the study of a very small number of parasite strains. Moreover, very few studies have evaluated in randomized controlled trials the efficacy of 0.25 mg/kg/day versus 0.5 mg/kg/day in real-life clinical endemic settings (John et al., 2012). A recent meta-analysis has concluded that there may be little or no difference in *P vivax* recurrences between the two regimens based on two studies, both conducted in India where *P. vivax* response to primaquine is believed to be intermediate between South-East Asia/Oceania isolates and temperate-Korean types ones (Milligan et al., 2020; Baird 2019). There is clearly a need for rigorous randomized controlled trials to evaluate the adequacy of those two regimens to prevent *P. vivax* relapses in endemic settings. A confounding factor that could affect the interpretation of the observed differences between tropical and temperate parasites is the unknown number of hypnozoites present in patient's liver: could a higher number of hypnozoites requires a higher dose of primaquine for effectively killing all parasites? As transmission intensity is typically higher in tropical areas than in temperate regions, one can assume that individuals from Chesson-type endemic areas harbor more hypnozoites than those from Korea-type ones. Under the current proposed mode of action of primaquine (production of H_2_O_2_ with cycling of OH-PQ m/O=PQm) and keeping the total dose effect in mind, this seems a plausible hypothesis, although it still has to be formally demonstrated.

## 6 Could resistance to primaquine emerge in *P. vivax* populations?

One factor could jeopardize the efforts towards vivax malaria elimination: the emergence of primaquine resistant parasites. Primaquine is a unique item in our toolbox against vivax malaria and the emergence of resistance would dramatically complicate eradication of *P. vivax* parasites. Mechanistically, given the MoA of primaquine, resistance of hypnozoites would require that parasites cope with oxidative stress. Such resistance mechanism is reminiscent of what is observed in *P. falciparum* resistance to artemisinin, also a drug that acts by generating ROS causing irreversible damage to the cell proteins (Wang et al., 2015; Ismail et al., 2016). The mechanisms of artemisinin resistance are not fully understood and involve several cellular and metabolic pathways including the implication of protein damage responses (Rocamora et al., 2018; Mok et al., 2015) and the entry into a dormant stage until the drug exposure has stopped (Witkowski et al. 2010, 2013; Teuscher et al., 2010). Whether *P. vivax* hypnozoites can also develop such strategies is unknown. Indeed, despite 70 years of using primaquine for *P. vivax* radical cure, *P. vivax* parasites resistant to primaquine have not been described. Emergence of resistance to any antimalarial drug seems to happen less frequently in *P. vivax* than in *P. falciparum* (possibly due to the greater reservoir of asymptomatic individuals and the earlier production of sexual parasites facilitating transmission) (Mueller et al., 2009). In addition, the biomass of hypnozoites exposed to the drug is probably quite low compared to blood stage parasites, decreasing the likelihood of selecting resistance. However, the selective pressure exerted by primaquine on hypnozoites would be very high as there is no escape possible for those hepatic forms and they either are killed or resist the treatment. We discuss here the factors that could, at least theoretically, lead to the emergence of primaquine resistance in *P. vivax* parasites (summarized in Fig. 1).

If the differences in primaquine susceptibility described in earlier studies are genuine, they suggest that there exist in the parasite population genetic polymorphisms underlying drug susceptibility that could more easily be selected for by sub-optimal treatment (natural selection on standing variation). In addition, many *P. vivax* parasites are likely currently exposed to sub-therapeutic doses of primaquine, due to incomplete radical cure treatment associated with poor compliance, or to standard regimen poorly suited to a host's decreased metabolism, which could facilitate selection of resistant alleles. Tafenoquine being slowly eliminated and remaining for longer in patients' blood would probably exert an even higher selection of those resistant alleles. In this regard, the deployment of single-low dose of primaquine against *P. falciparum* gametocytes could further increase exposure of *P. vivax* dormant liver parasites to the active metabolites in sub-therapeutic concentrations. Although the dose of primaquine for transmission-blocking is too low (1/14th to 1/28th of doses used for radical cure) to kill even fully susceptible hypnozoites, it could, in theory, trigger a response of hypnozoites against oxidative stress and perhaps predispose parasites to withstand hypnozoitocidal doses of primaquine. Drug-drug interactions, whether with blood-stage antimalarials (such as lumefantrine) or other medication also processed through the enzymes involved in primaquine metabolism could also, in theory, lead to sub therapeutic exposure of hypnozoites. Whether those factors could lead to resistance is currently unknown but given the unique position of primaquine in our antimalarial drug portfolio and the key importance of relapse in vivax malaria, emergence of primaquine-resistant parasite would be dramatic for our elimination efforts and needs to be considered.

## 7 Conclusions and future directions

Among all the factors discussed in this article, the most important one that can be leveraged to prevent emergence of resistance to primaquine (as for any antimicrobial drug) is to treat infections with the appropriate dose. It is quite astonishing that after decades of primaquine usage, we still do not know the optimal regimen to kill hypnozoites in many endemic areas. Low dose primaquine (3.5 mg/kg) spread over 14 days is a regimen commonly used to mitigate risks of hemolysis. With the advent of point-of-care G6PD quantitative tests, a higher dose of primaquine spread over a shorter duration could perhaps overcome issues related to polymorphism in cytochromes or high hypnozoite burden which could prevent emergence of resistance. Nevertheless, as mentioned above, there is currently no evidence of resistance to primaquine in *P. vivax* populations. However, if resistance would emerge, we would probably not be able to rapidly identify these parasites before they became a major public health issue. Indeed, there is no straightforward, easily implementable and unambiguous way to evaluate parasite susceptibility to primaquine. In vivo therapeutic efficacy studies evaluate the proportion of individuals remaining parasite-free after primaquine treatment during their follow-up that should last at least 6 months and up to one year (John et al., 2012). Even if confounders such as supervised primaquine administration or human enzyme polymorphism are controlled for, it is difficult to determine if parasite recurrence is due to higher tolerance to primaquine or to a reinfection by a new mosquito inoculation. Only relocating patients to a no-transmission area during the entire duration of the follow-up could mitigate the risk of reinfection, but this is obviously not an option for routine surveillance of primaquine efficacy in endemic countries. The only option in endemic settings would be to evaluate the proportion of recurrences following primaquine treatment on a large number of individuals year after year. Increase in these proportions could be a warning sign of declining efficacy of primaquine (assuming transmission intensity does not increase as well). In vitro assays or humanized mouse models to evaluate susceptibility of parasites to primaquine are alternatives to in vivo therapeutic efficacy studies greatly reducing the number of possible confounding factors. However, such liver-stage assays for *P. vivax* have only been developed in the past few years and present a number of challenges that prevent them from being used widely for routine characterization of clinical isolates. Whether employing human primary hepatocytes or humanized mouse (which harbors in vitro and in vivo features), they require sporozoites obtained following mosquito feeding on infected patients and come at a level of technical skills quite prohibitive for most National Malaria Control Programs of endemic countries (Mikolajczak et al., 2015; Roth et al., 2018; Gural et al., 2018).

In addition to the factors known to affect primaquine efficacy described in this paper, some questions remain open and could have implications on primaquine efficacy. Those mainly relate to the hypnozoites themselves whose biology is poorly understood. Hypnozoites are commonly regarded as a homogenous population of dormant, inactive cells. This view might be over simplistic and perhaps those cells are not as dormant as we think they are. Interestingly, in humanized mouse supporting *P. vivax* liver stage development as well as in in vitro culture of *P. vivax* liver stages using human primary hepatocytes, it was shown that the size of hypnozoites increased over time as well as contained an increasing number of apicoplasts compared to early hypnozoites, suggesting that some maturation and development may occur (Mikolajczak et al., 2015; Gural et al., 2018). This has implications for primaquine efficacy and it is currently unknown if all hypnozoites are affected by the drug similarly across this possible development, or if at some point of this maturation they are able to better cope with oxidative damages by the drug. Similarly, hypnozoites are commonly viewed as parasites infecting a homogenous population of host cells, hepatocytes. However, the liver is a complex organ with 3D architecture leading to zonation, mainly defined according to the distance of hepatocytes to the vascular system irrigating the liver (Lindros 1997). Zonation affects a vast number of cellular functions including drug metabolism and it is unknown whether all hypnozoite-infected hepatocytes have similar cytochrome activities leading to similar concentration of active metabolites following primaquine treatment. Further understanding of the hypnozoite biology is necessary to provide answers to those questions and in that regard, the in vitro liver-stage culture and humanized mouse models will be instrumental in improving our basic knowledge of *P. vivax* response to primaquine.

## Declaration of competing interest

We declare no conflict of interests.
